# Elderly Patients with Locally Advanced and Unresectable Non-Small-Cell Lung Cancer May Benefit from Sequential Chemoradiotherapy

**DOI:** 10.3390/cancers13184534

**Published:** 2021-09-09

**Authors:** Magdalena Zaborowska-Szmit, Marta Olszyna-Serementa, Dariusz M. Kowalski, Sebastian Szmit, Maciej Krzakowski

**Affiliations:** 1 Department of Lung Cancer and Thoracic Tumors, Maria Sklodowska-Curie National Research Institute of Oncology, 02-781 Warsaw, Poland; m.zaborowska3@gmail.com (M.Z.-S.); martaolszyna@gmail.com (M.O.-S.); Dariusz.Kowalski@pib-nio.pl (D.M.K.); Maciej.Krzakowski@pib-nio.pl (M.K.); 2 Department of Pulmonary Circulation, Thromboembolic Diseases and Cardiology, Centre of Postgraduate Medical Education, European Health Centre, 05-400 Otwock, Poland

**Keywords:** lung cancer, locally advanced, unresectable, sequential chemoradiotherapy, concurrent chemoradiotherapy, performance status, comorbidities, elderly, survival, prognosis

## Abstract

**Simple Summary:**

The combination of chemotherapy and radiotherapy, compared with radiotherapy alone, reduces the risk of local disease recurrence and the risk of distant metastases in patients with locally advanced unresectable non-small-cell lung cancer. Concurrent chemoradiotherapy is the most effective but also has the highest risk of toxicity. Older patients often have comorbidities and a reduced cardio-pulmonary capacity; therefore, they are less often qualified for concurrent chemoradiotherapy due to the predicted too high toxicity. The study documents the sense of considering sequential chemoradiotherapy in the elderly, regardless of whether they are in a good performance status and how many concomitant diseases were recognized earlier in their history. Compared to younger patients, the elderly benefit more from sequential chemoradiotherapy, because with the same toxicity, complete response is achieved more often and distant metastases are less frequently observed, which translates into a significantly longer survival.

**Abstract:**

Concurrent chemoradiotherapy is recommended for locally advanced and unresectable non-small-cell lung cancer (NSCLC), but radiotherapy alone may be used in patients that are ineligible for combined-modality therapy due to poor performance status or comorbidities, which may concern elderly patients in particular. The best candidates for sequential chemoradiotherapy remain undefined. The purpose of the study was to determine the importance of a patients’ age during qualification for sequential chemoradiotherapy. The study enrolled 196 patients. Older patients (age > 65years) more often had above the median Charlson Comorbidity Index CCI > 4 (*p* < 0.01) and Simplified Charlson Comorbidity Index SCCI > 8 (*p* = 0.03), and less frequently the optimal Karnofsky Performance Score KPS = 100 (*p* < 0.01). There were no significant differences in histological diagnoses, frequency of stage IIIA/IIIB, weight loss, or severity of smoking between older and younger patients. Older patients experienced complete response more often (*p* = 0.01) and distant metastases less frequently (*p* = 0.03). Univariable analysis revealed as significant for overall survival: age > 65years (HR = 0.66; *p* = 0.02), stage IIIA (HR = 0.68; *p* = 0.01), weight loss > 10% (HR = 1.61; *p* = 0.04). Multivariable analysis confirmed age > 65years as a uniquely favorable prognostic factor (HR = 0.54; *p* < 0.01) independent of lung cancer disease characteristics, KPS = 100, CCI > 4, SCCI > 8. Sequential chemoradiotherapy may be considered as favorable in elderly populations.

## 1. Introduction

In patients diagnosed with stage IIIA or IIIB non-small cell lung cancer (NSCLC) who are beyond surgical treatment, radical treatment with chemoradiotherapy or radiotherapy alone is the treatment of choice. The results of meta-analyses indicate that the combination of chemotherapy and radiotherapy is more effective than radiotherapy alone [1]. Concurrent chemoradiotherapy is superior to sequential treatment but is associated with a higher risk of severe complications (esophageal, hematological, pulmonary toxicity) [2].

As part of the sequential or concurrent chemoradiotherapy, conventional fractionation is used to a total dose of 60–66 Gy in 30–33 fractions for the area of primary lesion in the lung and the metastatic hilum and mediastinal lymph nodes. The chemotherapy regimen should include the administration of 2–4 cycles with a platinum derivative. In patients who responded to the concurrent chemoradiotherapy, administration of 12-month consolidation immunotherapy with durvalumab is associated with a further significant reduction of the relative risk of disease progression and a significant extension of overall survival with good tolerance of the treatment [3,4].

The above-mentioned results of the PACIFIC study indicate that currently the best management in inoperable and locally advanced NSCLC is concurrent chemoradiotherapy and consolidation immunotherapy [5]. Daily practice shows that not all patients can receive such treatment. Many factors determine the choice of sequential treatment in some patients. Importantly, the predicted toxicity is lower for sequential chemoradiotherapy compared to concurrent therapy. The role of consolidation immunotherapy after sequential chemoradiotherapy remains unknown, providing the rationale for the planning of further studies PACIFIC-5 (among inclusion criteria: receipt of concurrent or sequential chemoradiation therapy), PACIFIC 6 (a Phase II, Open-Label, Multi-Centre, International Safety Study of Durvalumab Following Sequential Chemotherapy and Radiation Therapy in Patients with Stage III, Unresectable Non-Small Cell Lung Cancer).

Pan-Asian adapted ESMO Clinical Practice Guidelines [6] indicate that concurrent chemoradiotherapy is the method of choice for treatment of patients with NSCLC in stage IIIA and IIIB in the absence of surgical treatment [the highest level of recommendations I, A]. If concurrent therapy is not applicable for any reason, sequential chemotherapy followed by radical radiotherapy is an alternative [the same level of recommendations I, A]. Elderly patients with impaired performance status, significant weight loss or comorbidities are excluded from prospective clinical trials assessing concurrent chemoradiotherapy [7]. Additionally, more than half of patients with stage III NSCLC theoretically have no chance of qualifying for a concurrent chemotherapy due to older age or concomitant diseases [8]. One of the first randomized controlled trials in elderly patients diagnosed with unresectable locally advanced NSCLC was planned to compare radiotherapy alone with a concurrent chemoradiotherapy. Although the chemotherapy was based on carboplatin, the study was terminated prematurely due to severe toxicity, some treatment-related deaths were observed [9]. According to the National Cancer Institute data, older patients with stage III NSCLC receiving chemoradiotherapy are more likely to experience treatment toxicity, including treatment-related deaths, which translated into worse overall survival (OS) compared to younger patients [10].

The largest analysis of data from the National Cancer Database from 2003 to 2014 on older patients showed that the combination of radiotherapy and chemotherapy gives a better prognosis than radiotherapy alone. In addition, it has been shown that in the elderly, sequential chemoradiotherapy compared with concurrent chemoradiotherapy results in a significantly more favorable OS with a reduction in mortality by 9% [11].

When identifying positive prognostic factors for sequential treatment with chemotherapy and radiotherapy, it is worth considering how to maintain this treatment algorithm in selected patients with satisfactory treatment results, especially if they could not receive concurrent chemoradiotherapy due to advanced age, low performance status or comorbidities. The lack of uniform guidelines developed by scientific societies confirms the necessity of such analysis.

The main aim of the study was to determine the prognostic significance of age in patients with locally advanced NSCLC who underwent sequential chemotherapy in the absence of the possibility of using surgical treatment and concurrent chemoradiotherapy.

## 2. Results

**Figure 1:**
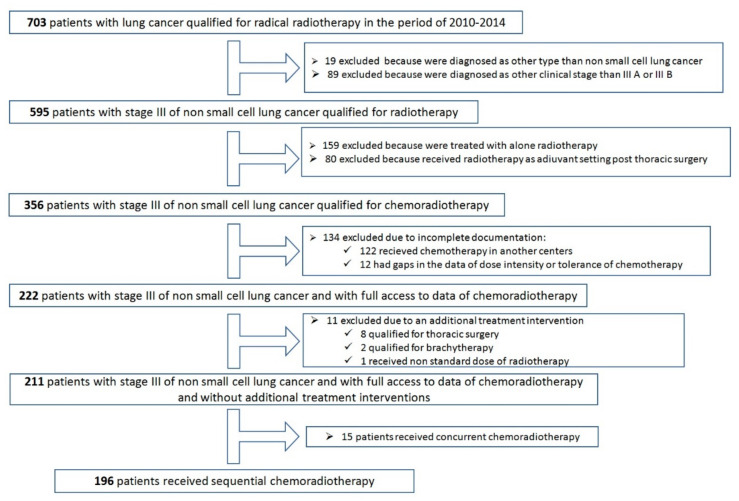
Flowchart presenting how data of patients undergoing radiotherapy were included in the study.

In accordance with the adopted algorithm, 196 patients who underwent sequential chemoradiotherapy were qualified for analysis (Figure 1).

The mean age of the patients was 60.52 ± 7.1 years. Among the histological diagnoses, squamous-cell carcinoma dominated in 91 (46.4%) of the patients, adenocarcinoma in 47 (24.0%), and other diagnoses in the remaining 58 (29.6%) patients. The clinical stage of stage IIIA and IIIB was established in 94 (48%) and 102 (52%) patients, respectively. Detailed patient characteristics are presented in Table 1.

Among 196 patients qualified for the analyzes determining the prognosis of patients undergoing sequential chemoradiotherapy, only one-third of patients (34.2%, 67 patients) had a very good performance status (100 according to the KPS scale) before treatment. The remaining patients had a lower KPS: 90 in 101 (51.5%) patients, 80 in 28 (14.3%) patients.

The following chemotherapy regimens were used: the PN regimen (cisplatin + vinorelbine) in 158 (80.1%) patients, the KN regimen (carboplatin + vinorelbine) in 18 (9. 2%) patients, the PG (cisplatin + gemcitabine) in nine (4.6%) patients, the PE regimen (cisplatin + etoposide) in five (2.6%) patients, PN/KN in two patients, PG/KG in one patient, KE in one patient, KG in one patient, and one patient received carboplatin + paclitaxel.

Finally, the chemotherapy regimen without cisplatin was administered in only 21 (10.7%) patients. More than half patients received two full cycles of chemotherapy: 106 (54.1%) patients. A large group received three cycles: 65 (33.2%) patients. Another two (1%) patients received three incomplete cycles. Unfortunately, eight (4.1%) patients received only one cycle due to poor tolerance, and 15 (7.7%) patients received four cycles of chemotherapy.

The median time between the end of chemotherapy and the start of radiotherapy was 28 days (4 weeks), the lower quartile was 19 days, and the upper quartile was 42 days (6 weeks).

The type and doses of radiation therapy were as follows:

➢ 5880 cGy—administered in 94 (48%) patients
➢ 6600 cGy—administered in 87 (44.4%) patients
➢ 6000 cGy—administered in 9 (4.6%) patients
➢ 6400 cGy—administered in 2 (1%) patients
➢ 6200 cGy—administered in 1 patient
➢ 5040 cGy—administered in 1 patient
➢ 5040 cGy + BOOST brachytherapy 7.5 Gy—administered in 1 patient
➢ 6200 cGy (4200 + BOOST to 6200)—administered in 1 patient

After radiotherapy, deterioration of KPS was observed in 53 (27%) patients, including a deterioration by 10 in 41 (20.9%) patients, by 20 in 10 (5.1%) patients and by 30 in two (1%) patients.

After a 6-week period from the end of radiotherapy, computed tomography imaging examinations were performed to assess radiological response to the chemoradiotherapy, and the following results were confirmed:

- complete response (CR) in 15 (7.7%) patients;
- partial response (PR) in 123 (62.8%) patients;
- stable disease (SD) in 44 (22.4%) patients;
- progressive disease (PD) in 14 (7.1%) patients.

After 10.6 years of follow-up, 168 (85.7%) patients died, and 28 (14.3%) patents were still alive. The median overall survival was 745 days (2years and 15 days) and the interquartile range was from 433 days (14.4 months) to 1475 days (4y ears and 15 days). The 5-year and 10-year survival rate was 19.9% and 12. 2%, respectively. During clinical follow-up, most of the patients experienced lung cancer disease progression, the characteristics of which are presented in Table 2. At the end of follow-up, 20 (10.2%) patients had no sign of cancer disease progression and no need to re-treat cancer. The median PFS was 390 days (13 months), the range between quartiles was 269 days (9months) to 889 days (29.6 months). The 5-year and 10-year progression-free survival rate was 13.3% and 7. 25%, respectively.

During the observation of the study population, new cases of other independent neoplasms were also found in nine (4.6%) patients. The diagnoses included two cases of pancreatic cancer, and one case each of breast cancer, gastric cancer, laryngeal cancer, esophageal cancer, renal pelvis cancer, melanoma, and the second primary lung cancer.

The analysis of the survival time showed that distant metastases (HR = 1.66; 95% CI: 1. 22–2. 26, *p* < 0.01), especially CNS metastases (HR = 1.8; 95% CI: 1. 21–2.68; *p* < 0.01) and hepatic metastases (HR = 1.93; 95% CI: 1.01–3.67; *p* < 0.05) had a negative impact on OS. Local recurrence, lymph node metastases, metastases in the second lung, pleural effusion, bone metastases did not have a significant prognostic significance for OS.

**Figure 2:**
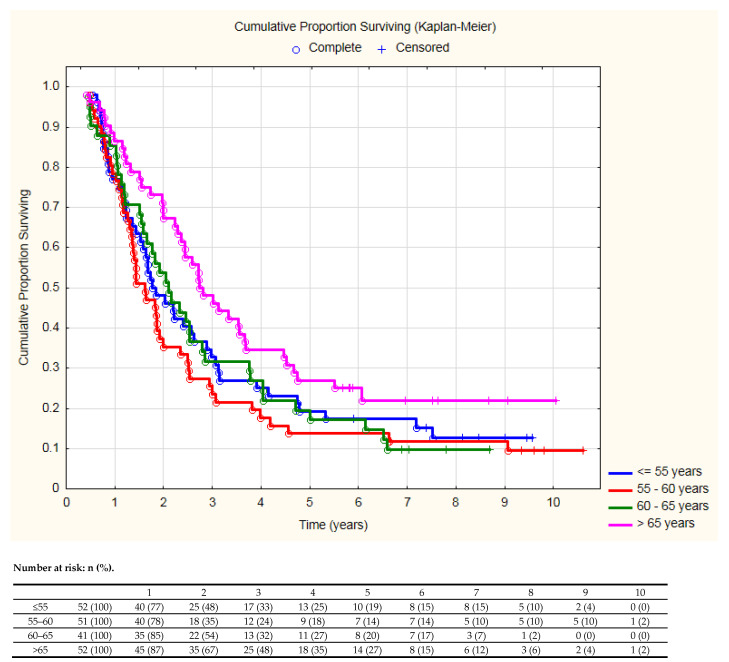
Overall survival in relation to quartiles of patients’ age (log-rank test *p* = 0.04).

According to the adopted methods, when the quartile values were used to assess the relationship between age and OS, it turned out that the age of patients in the upper quartile (over 65 years) differed significantly in OS compared to the younger groups (Figure 2).

**Figure 3:**
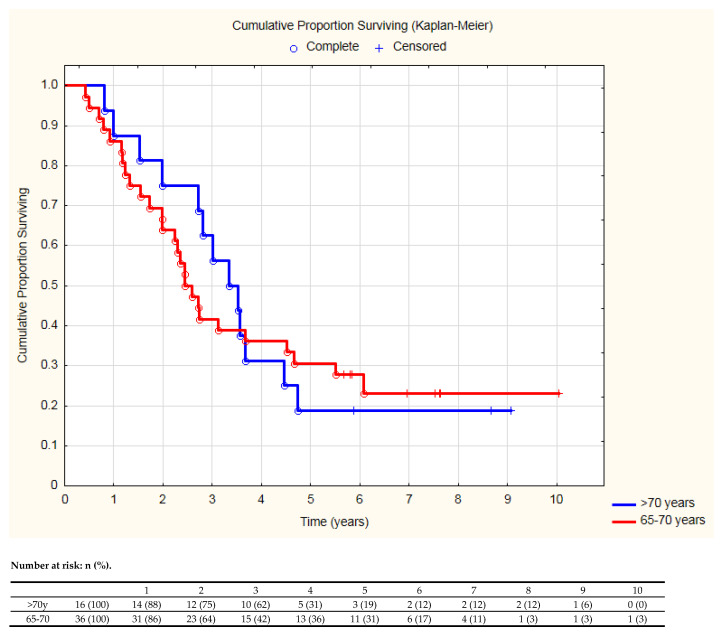
The comparison of overall survival between patients aged 65–70 and >70 years (log-rank test *p* = 0.91).

In the group of 52 older patients (over 65 years of age) there were 36 aged >65 and ≤70, and 16 patients over 70 years of age (including two patients over 75 years). The direct comparison of the survival curves of patients aged 65–70 and >70 years did not show a significant difference for OS (Figure 3).

In univariable analysis of Cox proportional hazards model among the baseline demographic patients’ characteristics or when defining the lung cancer disease itself, the following were significant: age over 65 years versus younger (HR = 0.66; 95% CI: 0.46–0.94; *p* = 0.02), disease stage IIIA versus IIIB (HR = 0.68; 95% CI: 0.50–0.92; *p* = 0.01), weight loss of more than 10% versus less (HR = 1.61; 95% CI: 1.03–2.53, *p* = 0.04). In multivariable analysis, an age over 65 was revealed to be the only significant favorable prognostic factor (HR = 0.54; 95%CI: 0.36–0.83; *p* < 0.01) independent of characteristics of lung cancer disease, good performance status or number of comorbidities evaluated by CCI or SCCI (Table 3).

The comparison of the baseline characteristics of older and younger patients showed that the elderly were significantly more likely to be obese and had more significant comorbidities as expressed by higher CCI or SCCI (Table 4). On the other hand, older patients were less likely to have a good performance status defined as KPS 100. There were no differences in terms of histological diagnoses, stage IIIA/IIIB, weight loss, level of smoking addiction.

**Figure 4:**
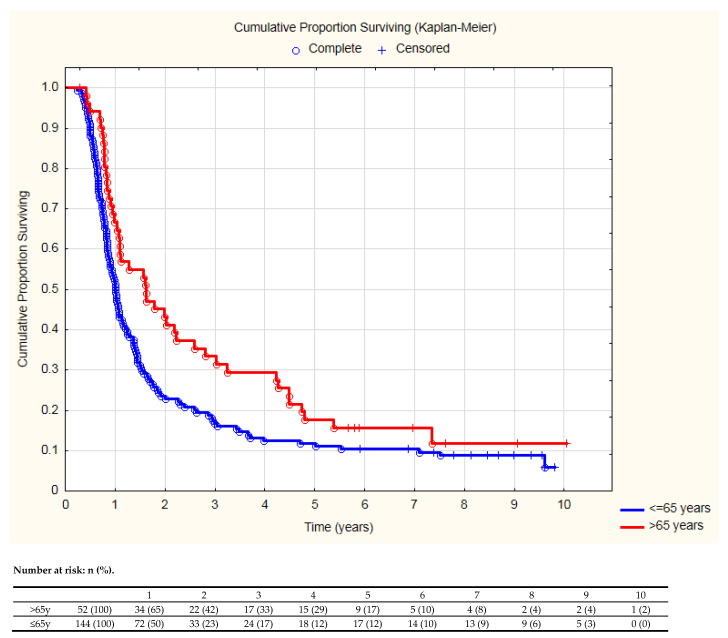
Progression free survival in older and younger patients (log–rank test *p* = 0.02).

The comparison of the sequential chemoradiotherapy characteristics and effectiveness between older (>65 years) and younger patients, revealed that elderly patients received chemotherapy without cisplatin more often (Table 5). Older patients more often had complete response recognized 6 weeks after radiotherapy, and less frequently experienced a deterioration of their performance status. Serious treatment complications occurred with similar frequency in both age groups. Elderly patients experienced distant metastases less frequently and had significantly longer PFS (Figure 4).

Significantly longer PFS had to determine longer OS in elderly patients because there were no differences in the frequency of systemic therapy after cancer progression, either in the frequency of chemotherapy, or immunotherapy or targeted therapy. Additionally, there was also no difference in the number of lines of chemotherapy (Table 6).

## 3. Discussion

The results of the study indicate that patients over 65 years had significantly better OS after sequential chemoradiotherapy compared to younger patients, despite the more frequent coexistence of comorbidities (higher CCI or SCCI) and less frequently they maintained a good performance status (as KPS 100). It seems that sequential chemoradiotherapy may be a valuable treatment in elderly patients with locally advanced NSCLC. We demonstrated that, despite the less intensive treatment, older patients achieved complete responses more often, experienced distant metastases less frequently, and PFS was significantly longer compared to younger patients. It is very important that the risk of significant complications was comparable in older and younger patients. The risk of deterioration of performance status, and thus the risk of deteriorating quality of life, was lower in the elderly compared to the younger ones. The results of the treatment were independent of the initial clinical stage of the lung cancer disease (stage IIIA vs. IIIB).

NSCLC is a neoplasm with moderate radiosensitivity. Radiotherapy alone does not bring a satisfactory benefit in the treatment of patients with a stage III diagnosis because the median OS in prospective studies did not exceed 26 months [12,13]. After radiotherapy, a high risk of distant metastases and limited locoregional effectiveness is observed. The main goal of combining chemotherapy and radiotherapy is to increase the chance of local control of unresectable disease as well as to reduce the risk of distant metastases. The combination therapy includes the use of additive local effects of both methods and the destruction of subclinical metastases outside the irradiation field.

Concurrent chemoradiotherapy is currently considered the standard of care for patients with locally advanced and inoperable NSCLC. Sequential chemoradiotherapy is recommended for patients in whom the risk of toxicity during concurrent treatment is too high and in cases with too large a tumor volume. Since these criteria can be very subjective, an observational study was conducted to determine the percentage of patients with locally advanced NSCLC who are actually eligible for both treatment methods and to identify factors which determined the choice of treatment [14]. The study included data from three independent registries: Belgian Cancer Registry (BCR), Netherlands Cancer Registry (NCR), Dutch Lung Cancer Audit–Radiotherapy (DLCA–R), including 350, 780, and 428 patients, respectively. Among NSCLC patients with stage III, more than half of the patients in the Netherlands (55%) and more than one-third in Belgium (35%) received concurrent treatment. In both populations, older age and more advanced N trait were significantly associated with the preference for sequential therapy. Interestingly, the overall cardio–pulmonary fitness, comorbidities or tumor volume were not significant. Despite the strong limitations of our study, such as its retrospective nature, disproportions in the number of patients below and above 65 years of age, and the random distribution of other factors, the demonstration that older age and the presence of the N3 feature were not associated with worsening of OS seems to be a clinically valuable observation.

It is assumed that the better tolerance of sequential chemoradiotherapy as compared to concurrent chemoradiotherapy results from the occurrence of side effects of chemotherapy and radiotherapy at different times, which translates into the possibility of carrying out the entire treatment in optimal doses and an acceptable total time of therapy (no significant interruptions due to toxicity). The disadvantage of sequential chemoradiotherapy is a prolongation of the total treatment time. Along with the number of chemotherapy cycles and the time between the end of chemotherapy and start of radiotherapy, cancer cell repopulation may be observed and the probability of radiation resistance may increase. In our study, this effect could have affected younger patients in whom efficacy of sequential chemoradiotherapy was significantly worse than in older patients.

The factors determining disqualification from concurrent chemoradiotherapy include advanced age (usually age > 75 years), significant comorbidities, high local advancement, weight loss >10% of the predicted value, and abnormal cardiopulmonary capacity. Unfortunately, the unfavorable regularity is that older patients usually have several comorbidities and a reduced performance status. Apart from the assessment of the performance status, the assessment of comorbidities turns out to be very useful in the assessment of prognosis [15]. ASCO recommends that in elderly patients a comprehensive geriatric evaluation should be used [16]. In this way, it is possible to identify fit elderly patients who may even benefit from concurrent chemoradiotherapy [17,18].

Various groups of experts recommend that patients without significant comorbidities are eligible for concurrent chemoradiotherapy [19]. For the purpose of objectivization, it is recommended to use the Charlson Comorbidity Index (CCI) or the simplified version—the Simplified Charlson Comorbidity Index (SCCI)—to assess the significance of comorbidities. The CCI is the most widely used scale for assessing comorbidities that determine prognosis [20]. The simplified comorbidity score (SCCI) for lung cancer prognosis was proposed by Colinet et al. [21]. It is a weighted sum of seven components: smoking, diabetes, kidney failure (7,5,4, points, respectively) and respiratory disease, cardiovascular disease, other previous cancer disease, alcoholism (one point each, respectively). The clinical value of SCCI has been tested in a study where the effectiveness of radiotherapy alone or combined with chemotherapy was compared in the elderly [22]. PFS and OS after chemoradiotherapy were significantly better in elderly patients with good performance status and SCCI < 10. Severe pulmonary toxicity was significantly more frequent in the elderly and fragile (*p* = 0.03). Pulmonary complications in grade three or higher were associated with worsening of OS (*p* = 0.04). In our observation, multi–morbidity defined as CCI> 4 (above the median) or SCCI > 8 (above the median) was not associated with a significant deterioration of OS, as demonstrated by both the univariable and multivariable analysis. Our results confirm that older patients (>65 years of age) regardless of their comorbidities may benefit significantly from sequential chemoradiotherapy.

However, there are no prospective studies that would unambiguously indicate at which value obtained in the CCI or SCCI or any other scale should patients be disqualified from concurrent chemoradiotherapy, and at which value they may be disqualified from sequential chemoradiotherapy. Although SCCI is more specific for lung cancer patients, our observation showed that the CCI differentiated the older from the younger in a more significant way than the SCCI. However, neither of the scales in the univariable or multivariable analysis turned out to be significant for the prognosis, even assuming values above the median in both of these scales (CCI > 4 and SCCI > 8).

The qualification of elderly NSCLC patients for optimal therapy is problematic regardless of the stage of the cancer disease [23]. In older patients, delay in subsequent stages of treatment is more often observed, the toxicity of therapy is greater, and the prognosis is unfortunately less favorable [24]. Most recommendations of scientific societies indicate the age of 75 to be the age limit for qualification for simultaneous treatment [25]. Such patients should be assessed according to the geriatric scales and the comorbidity index (CCI or SCCI), as well as cardiopulmonary and other organ capacity should be considered during qualification for sequential chemoradiotherapy, radiotherapy alone, or palliative therapy. In our study older patients more often had many comorbidities (CCI > 4 in 29.2% of the young versus 82.7% in the elderly, SCCI > 8 in 25.7% of the younger versus 42.3% in the elderly), and a lower performance status (KPS equal to 100 in 40.1% younger and in 15.4% older (Table 3). However, older age in the highest quartile of our population occurred as positive for OS after sequential chemoradiotherapy. That is a sufficient reason for sequential chemoradiotherapy to be considered in older patients.

Radical independent radiotherapy seems to be an accepted therapeutic choice in patients with locally advanced and inoperable NSCLC aged ≥75 years [26]. Randomized trials have shown that sequential chemoradiotherapy is superior to radiotherapy alone [27]. Although there are no clear data on the safety of sequential chemoradiotherapy in elderly patients, this therapeutic option is certainly better tolerated than concurrent chemoradiotherapy [28]. In retrospective analyses, older and younger patients achieved comparable OS after sequential chemoradiotherapy, but the risk of haematological and non-hematological complications was greater in the elderly under short-term follow-up [29,30]. This may be a clinical problem, if not for the prognosis, then for the quality of life. The demonstration in our study that elderly patients had a better OS without increasing toxicity in CTCAE grade 3/4 compared to younger patients, justifies considering sequential treatment in older patients.

The efficacy of concurrent chemoradiotherapy was analyzed in 66 patients aged 71–78 years with an increased risk of complications: performance status according to WHO 2–3, concomitant heart, lung or kidney diseases, and weight loss [31]. As part of chemotherapy, vinorelbine was used in combination with carboplatin (59 patients) or cisplatin but at a dose of 20 mg/m^2^ (only seven patients). Conventional radiotherapy up to a total dose of 63 Gy was used at the same time. A very high rate of significant toxicity was observed: anemia requiring transfusions in 26%, grade 3 neutropenia in 38% and grade 4 in another 4%, grade 3 thrombocytopenia in 12% and grade 4 in another 15% of patients. Among other complications oesophagitis was observed: grade 3 in 3% and grade 4 in 2%, pneumonia in grade 3 in 3%. Overall survival was 53%, 24%, and 8% at 12 months, 24 months, and 5 years of follow-up, respectively. Thus, the results were significantly worse than those observed in our study for sequential chemoradiotherapy.

In a randomized trial conducted by the Japan Clinical Oncology Group (JCOG0301), patients over 70 years of age with inoperable stage III NSCLC were recruited, and concurrent chemoradiotherapy was administered: irradiation (60 Gy) and a low dose of carboplatin (30 mg/m^2^ daily for 5 days a week, all for 20 days) [32]. The comparative arm had radioterapy alone. A total of 200 patients were enrolled (100 patients in each group). Median OS was 22.4 months and 16.9 months in favor of concomitant treatment (HR = 0.68, *p* = 0.02). More complications occurred with chemoradiotherapy—grade 3/4 hematological complications: leukopenia 63.5%, neutropenia 57.3%, thrombocytopenia 29. 2%. Such complications were not noted in the group receiving radiotherapy alone. Grade three infections were more frequently observed with chemoradiotherapy (12.5% vs. 4.1%). In contrast, grade 3/4 of pneumonia and other pulmonary toxicities occurred with comparable frequency in both arms. There were seven treatment-related deaths: three (3%) in patients receiving chemoradiotherapy and four (4%) in the group with radiotherapy alone. Thus, the benefit in OS was over 5months, but the risk of toxicity in older patients was clearly greater. In our study, we did not observe treatment-related deaths. The median OS was 24.5 months and was also more favorable than in the cited study, even though we used sequential treatment (not concurrent like in the cited study).

Experts agree that the elderly may even be eligible for concurrent chemoradiotherapy, if they remain fit and have no significant comorbidities. Our own data allow us to conclude that elderly patients (especially those over 65 years of age) may be good candidates for sequential treatment even in the presence of significant cardiovascular or other diseases. Our results seem to be promising in relation to data discussed above (Table 7).

Oncologists focus on prognostic factors directly related to cancer disease. There is an ongoing discussion regarding the differences in prognosis in stage III of NSCLC depending on the histological type (mainly between squamous-cell carcinoma and other types). In the case of squamous-cell carcinoma, it seems that local recurrences are more frequently observed, with distant metastases and a worse prognosis in other histological types [33,34]. In our study, the better prognosis for older patients, however, was not due to the higher incidence of squamous-cell carcinoma, because the elderly had similar histological characteristics to younger patients. Moreover, we know that the presence of the N3 trait is associated with a higher risk of distant metastases [35,36]. In our observation, older age turned out to be a favorable prognostic factor, regardless of stage IIIA, feature N3 or T4 in multivariable analysis. There were also no differences in the incidence of stage IIIA, T4 and N3 traits in the older population compared to the younger.

The addiction to smoking is directly related to the risk of lung cancer but is also a strong risk factor for atherosclerosis and obstructive pulmonary disease and, therefore, can cause cardiopulmonary failure. [37]. In our own observation, a heavy addiction to smoking (over 50 packyears) affected the elderly and younger patients to the same extent, and it did not show a significant effect on OS in the multivariable analysis.

A prognostic factor indicating a worse prognosis in patients with NSCLC is the loss of body weight above 10% of the predicted value within the three months preceding the start of anticancer treatment [38]. Our study by a multivariable analysis showed that weight loss of ≥10% in elderly patients lost its significant prognostic value.

Experts recommend the use of cisplatin-based regimens as part of chemoradiotherapy. On the other hand, an Asian study dedicated to elderly patients undergoing chemoradiotherapy found a benefit was obtained with carboplatin-based chemotherapy [32]. There is agreement that carboplatin-based CT regimens should be used in patients with contraindications for the use of cisplatin due to coexisting diseases. The concern about significant cardiovascular complications related to cisplatin seems to be justified [39]. Our study showed that such careful handling may be prognostic because our elderly population lived significantly longer regardless of the more frequent use of cisplatin-free chemotherapy regimens.

In the local advancement stage (CS III), qualifying NSCLC patients for an appropriate treatment method should consider the analysis of prognostic and predictive factors, where their effectsin patients with locally advanced NSCLC havenot been sufficiently documented so far.

The effectiveness of chemoradiotherapy is constantly improving. This is possible thanks to the increasingly precise diagnosis of the cancer stage and precise qualification for radical treatment. Moreover, radiotherapy is becoming more technologically advanced and more effective [40].

The median age for newly diagnosed lung cancer patients is just over 70 years, while studies comparing sequential with concurrent chemoradiotherapy include a very small percentage of the elderly. Another problem is related to comorbidities that occur more frequently among the elderly, which also have an impact on OS because of a high risk for comorbidity-related events. Personalized management of elderly patients should be based on the knowledge of prognostic factors, which is essential in modern thoracic oncology.

ASCO reminds us that the ideal concurrent chemotherapy regimen has not been determined [41]. For patients who cannot tolerate concurrent chemoradiotherapy, sequential chemotherapy followed by radical radiation may be an option. However, to date the predictive factorsfor the intolerance of concurrent chemoradiotherapy have been undefined and the best candidates for sequential chemoradiotherapy remain unknown. ASCO believes radiotherapy alone may be used as definitive radical treatment for patients who are ineligible for combined-modality therapy due to poor performance status, comorbidities, or extensive weight loss.

The 2010 meta-analysis is consistently cited as the background of the evidence showing the advantage of concurrent chemoradiotherapy over sequential therapy [2]. Meanwhile, our own data regarding OS clearly show that the results of sequential treatment may be better than the historical ones. This is due to the aforementioned improvement in the assessment of NSCLC advancement and an increase in the effectiveness of radiotherapy. However, there is one more important comparative aspect. Updated results of the PACIFIC study confirmed a 29% reduction in the risk of death thanks to treatment with durvalumab [42]. The median OS was 47.5 months for the durvalumab arm versus 29.1 months for the comparator arm with concurrent chemoradiotherapy. Comparing the OS in the current study with sequential chemoradiotherapy, it turns out that OS was better in the first year, comparable after two years, and less favorable from the third year of follow-up. This result is certainly influenced by coexisting cardiological and internal diseases, which the patients in the PACIFIC study did not have. This requires further research, with a particular focus on not only the age of patients, but also comorbidities, their control, and treatment effectiveness.

## 4. Limitations of the Study

The presented results come from a retrospective exploratory analysis and may be the basis for hypotheses to be verified in future prospective trials. We collected information retrospectively after the occurrence of events, the analysis of which was not planned a priori, there was a possibility of omitting important information. Multiple a posteriori comparisons with the adopted level of statistical significance *p* = 0.05 could cause some significant results to be obtained by chance. Nevertheless, the prognostic significance of the key outcome for the age of patients was verified by multivariable analysis.

The results presented are also subject to selection errors. A significant problem is the representativeness of the studied group. The average age of the patients included in the analyzes was only 60.5 years, compared to significantly higher age in people diagnosed with lung cancer in the world. The number of patients in our analysis was relatively small, which is related to the single-center nature of the study and including only patients with full clinical data available in the documentation. On the other hand, the study comes from the largest cancer center in Poland, where therapeutic decisions are made based on multidisciplinary teams.

Our study showed that the population of patients with inoperable locally advanced lung cancer is very heterogeneous. The mere comparison of older with younger patients reveals significant differences in morbidity, BMI, and fitness status etc. Univariable analyzes can only produce random results. Our multivariable analysis shows how important the interactions between the different features are and how important is to understand the issue of age in oncology. It is true that the multivariable analysis confirmed that the elderly had the lowest mortality risk, but regardless of this, there were interactions between other traits, which can be seen in the HR values, e.g., for T4 feature. However, apart from older age, no other parameter gave a statistically significant impact on OS. Thus, it can be concluded that elders may benefit from sequential chemoradiotherapy.

## 5. Materials and Methods

The inclusion criteria focused on patients with locally advanced and inoperable NSCLC who were qualified for radical radiotherapy in the National Institute of Oncology —National Research Institute in Warsaw (Poland). The clinical advancement of NSCLC was assessed according to the 7th edition of TNM, in which the clinical stage III (CS III) was divided into two groups: CS IIIA and CS IIIB.

As part of staging, a CT of chest and epigastrium and an MRI/CT of the CNS were performed. All patients had a PET–CT examination performed prior to chemoradiotherapy initiation. Invasive diagnosis of the N feature was performed in patients with suspected N2/3 feature based on the results of PET–CT or CT.

In accordance with the adopted internal standards, the sequential use of chemotherapy and radiotherapy was dedicated to elderly patients, with a lower degree of fitness, with concomitant diseases but without contraindications to the administration of chemotherapy, with an estimated relative high tumor mass (i.e., with borderline indications for radical treatment) and in patients who did not consent to concurrent chemoradiotherapy due to fear of toxicity.

The ARIA Oncology Information System used in the Department of Radiotherapy was used to identify patients. The following factors were used as search criteria:

(1) diagnosis of C34 according to ICD 10;
(2) number of fractions of radiotherapy—21, 30 or 33;
(3) the period of using radiotherapy: 2010–2014.

Data on chemotherapy were obtained from the CLININET hospital system, chemotherapy always preceded radiotherapy, and a first patient was enrolled in July 2009 and a last one in October 2014. The principles of selecting patients for analysis are presented in Figure 1. No patient was lost from follow-up, that was a prerequisite for inclusion in this retrospective analysis: the patient either reported to scheduled follow-up visits or was reported dead. No one died while on active treatment or within the 6 weeks to first follow-up when CT assessment was performed on the chest with epigastrium. Then, the patients reported for follow-up visits every 3 months for the first 3 years of follow-up, every 6 months for the next 2years, and then every year. A CT scan was performed every 6 months for the first 5 years, and then every year. If the patient did not come to the control visit, we checked the date of death. Data on deaths were obtained from the National Cancer Registry.

In the analysis of predictors for OS, apart from age, the following factors were taken into account:

A—assessed before anticancer treatment:

- patient-related: gender, BMI/obesity, smoking, fitness status, weight loss within the 3 months prior to starting chemotherapy;
- cancer-related: stage IIIA vs. IIIB, feature N 3, feature T 4;
- comorbidities-related expressed by the Charlson Comorbidity Index (CCI) and the Simplified Charlson Comorbidity Index (SCCI).

B—assessed during or after treatment:

- occurrence of adverse events grade 3 or higher according to CTCAE;
- deterioration of performance status confirmed by the Karnofsky Performance Score (KPS);
- response to treatment according to RECIST 1.1, assessed 6 weeks after the end of radiotherapy;
- type of the cancer disease progression (recurrence or distant metastases);
- progression-free survival (PFS)

## 6. Statistical Analysis

When characterizing patients in terms of features expressed on a nominal scale, the number and percentage of the total were used. Particular features related to the baseline characteristics of patients and lung cancer disease, as well as chemoradiotherapy (including the effectiveness and safety) and subsequent systemic anticancer therapies were compared between older and younger patients using the chi2 test with a possible Yates’ correction.

The primary goal was to define OS, which was the time from the start of chemotherapy to the moment of death from any cause. Age quartiles of the study population were used to evaluate OS differences according to Kaplan–Meier survival curves. Cox proportional hazards analysis was used to assess the significance of all available potential prognostic factors—univariable and multivariable models were used.

## 7. Conclusions

Our study proved that elderly patients may be considered for sequential chemoradioterapy. The unique value of the study is a long-term follow-up (10 years) with assessment of predictors of the outcome of stage III unresectable NSCLC patients in the pre-immunotherapy era. Authors believe such data may be useful to plan individualized optimal model of treatment this heterogenous group of lung cancer patients in current time of immuno-oncology.

## Figures and Tables

**Table 1:** Characteristics of patients qualified for sequential chemoradiotherapy.

Parameters	Number (%)
Sex	Female—69 (35.2)
	Male—127 (64.8)
Age	
≤65	144 (73.5)
>65	52 (26.5)
BMI (kg/m^2^) <25 kg/m^2^	77 (39.3)
Overweight (BMI: ≥ 25 and <30 kg/m^2^)	68 (34.7)
Obesity (BMI ≥ 30kg/m^2^)	51 (26)
Lack of weight loss	107 (54.6)
Weight loss <10%	65 (33. 2)
Weight loss ≥10%	24 (12. 2)
Pathology	
Squamous-cell carcinoma	91 (46.4)
Adenocarcinoma	47 (24.0)
Other types	58 (29.6)
Clinical Stage	III A–94 (48)
	III B–102 (52)
TNM	
TX	2 (1)
T0	2 (1)
T1	14 (7.1)
T2	70 (35.7)
T3	44 (22.4)
T4	64 (32.7)
N0	13 (6.6)
N1	9 (4.6)
N2	154 (78.6)
N3	20 (10. 2)
Declared smoking status	
never smokers	16 (8.16)
<20 pack years	41 (20.92)
20–50 pack years	94 (47.96)
≥50 pack years	45 (22.96)
Karnofsky performance status (KPS)	
100	67 (34.2)
90	101 (51.5)
80	28 (14.3)
Charlson Comorbidity Index (CCI)	
≤3	56 (28.6)
4	55 (28.1)
5	44 (22.4)
≥6	41 (20.9)
Simplified Charlson Comorbidity Index (SCCI)	
≥7	72 (36.7)
8	65 (33.2)
≥9	59 (30.1)

**Table 2:** Characteristics of lung cancer disease progression after sequential chemoradiotherapy.

Type of Cancer Disease Progression	Number (%)
Local recurrence	87 (44.4%)
Distant metastases	81 (41.3%)
nodal metastases (N feature)	23 (11.7%)
pleural effusion	12 (6.1%)
metastases to the second lung	21 (10.7%)
metastases to the central nervous system	32 (16.3%)
bone metastases	11 (5.6%)
liver metastases	10 (5.1%)
adrenal metastases	6 (3.1%)

**Table 3:** Baseline preexisting predictors of OS.

Patients with a Possible Predictor Compared to Patients without Such Feature	Univariable			Multivariable		
	HR	95% CI	*p*-Value	HR	95% CI	*p*-Value
Women	0.78	0.56–1.08	0.13	0.79	0.56–1.11	0.18
Age > 65 years	0.66	0.46–0.94	0.02	0.54	0.36–0.83	<0.01
Obesity (BMI ≥ 30kg/m^2^)	0.92	0.65–1.31	0.64	0.95	0.65–1.4	0.8
Squamous–cell carcinoma	0.99	0.73–1.34	0.94	0.95	0.64–1.40	0.78
Adenocarcinoma	0.81	0.56–1.16	0. 24	0.84	0.54–1.32	0.45
Stage IIIA vs. IIIB	0.68	0.50–0.92	0.01	0.7	0.47–1.06	0.09
T4	1.12	0.81–1.54	0.5	0.96	0.63–1.46	0.85
N3	1.10	0.68–1.80	0.69	1.02	0.57–1.85	0.94
Smoking ≥50 pack years	0.90	0.62–1.30	0.56	0.85	0.58–1. 25	0.41
Weight loss ≥10%	1.61	1.03–2.53	0.04	1.50	0.91–2.48	0.11
Performance status KPS = 100	0.96	0.69–1.32	0.79	0.89	0.62–1. 26	0.5
Charlson Comorbidity Index (CCI) >4	1.12	0.83–1.52	0.46	1.45	0.97–2.15	0.07
Simplified Charlson Comorbidity Index (SCCI) >8	1.0	0.72–1.39	0.99	0.9	0.62–1.32	0.59

**Table 4:** The comparison of baseline characteristics between older and younger patients.

		≤65 Years (144 Patients)	>65 Years (52 Patients)	*p*-Value
Demographic	Female	52 (36.1%)	17 (32.7%)	0.7
	Obesity (BMI ≥30 kg/m^2^)	32 (22.2%)	19 (36.5%)	0.04
Pathology	Squamous–cell carcinoma	64 (44.4%)	27 (51.9%)	0.35
	Adenocarcinoma	35 (24.3%)	12 (23.1%)	0.86
	Other types	45 (31.3%)	13 (25%)	0.4
Primary tumor and mediastinal nodes stage	Clinical stage IIIA	67 (46.5%)	27 (51.9%)	0.5
	T4	43 (29.9%)	21 (40.4%)	0.17
	N3	14 (9.7%)	6 (11.5%)	0.7
Smoking	≥20 pack years	104 (72.2%)	35 (67.3%)	0.5
	≥50 pack years	33 (22.9%)	12 (23.1%)	0.98
Weight loss	Not observed	79 (54.9%)	28 (53.9%)	0.9
	≥10%	20 (13.9%)	4 (7.7%)	0.36
Performance status KPS = 100		59 (40.1%)	8 (15.4%)	<0.01
Comorbidities	Charlson Comorbidity Index (CCI) >4	42 (29.2%)	43 (82.7%)	<0.01
	Simplified Charlson Comorbidity Index (SCCI) >8	37 (25.7%)	22 (42.3%)	0.03

**Table 5:** The comparison of parameters related to chemoradiotherapy between older and younger patients.

		≤65 Years (144 Patients)	>65 Years (52 Patients)	*p*-Value
Chemotherapy	Cisplatin + vinorelbine	121 (84%)	37 (71. 2%)	0.04
	Regimen without cisplatin	9 (6.3%)	12 (23.1%)	<0.01
Longer time between the end of chemotherapy and start of radiotherapy (defined as > 42 days/6 weeks)		31 (21.5%)	14 (26.9%)	0.4
Deterioration of performance status at least by 10 points (KPS)		45 (31.3%)	8 (15.4%)	0.03
Occurrence of complications grade 3/4 according to CTCAE		45 (31.3%)	22 (42.3%)	0.15
Response according to RECIST evaluated 6 weeks after the end of radiotherapy	CR	7 (4.9%)	8 (15.4%)	0.01
	PR	95 (66%)	28 (53.9%)	0.12
	SD	30 (20.8%)	14 (26.9%)	0.37
	PD	12 (8.3%)	2 (3.9%)	0.45
Type of cancer disease progression	Local progression	59 (40.1%)	28 (53.9%)	0.11
	Distant metastases	66 (45.8%)	15 (28.9%)	0.03
Secondary cancer disease		7 (4.9%)	2 (3.9%)	0.93

**Table 6:** The comparison of systemic subsequent therapies between older and younger patients with progression after sequential chemoradiotherapy.

Subsequent Systemic Therapies	≤65 Years (144 Patients)	>65 Years (52 Patients)	*p*-Value
Chemotherapy	69 (47.9%)	24 (46. 2%)	0.83
Number of lines of chemotherapy			
1.	43 (29.9%)	20 (38.5%)	0.31
2.	17 (11.8%)	4 (7.7%)	
3.	7 (4.9%)	0	
4.	2 (1.4%)	0	
Immunotherapy	4 (2.8%)	2 (3.8%)	0.93
Targeted therapy	2 (1.4%)	1 (1.9%)	
Anti–EGFR	1	1	
Anti–ALK	1	0	0.7

**Table 7:** The key studies cited in the discussion.

First Author of the Study	Design of the Study	Main Result or Conclusion
Atagi S. [9]	Patients: 71 years of age or older. Randomization: radiotherapy alone vs. chemoradiotherapy (concurrent use of carboplatin)	Terminated due to treatment-related deaths.
Stinchcombe T.E. [10]	16 phase II or III trials of concurrent chemoradiotherapy	Elderly patients under concurrent chemoradiotherapy had unbeneficial OS, higher rate of toxicity (including death).
Miller E.D. [11]	Patients: elderly (≥70 years old). Comparative effectiveness study of radiation therapy versus chemoradiation	Sequential chemotherapy and radiation resulted in a 9% mortality reduction in comparison to concurrent treatment.
Lee J.H. [22]	Patients: aged 70 years or more. Treatment: radical radiotherapy with or without chemotherapy	Simplified comorbidity score (SCS) was the independent prognostic factor for OS. Chemoradiotherapy was superior to radiotherapy in the fit elderly with SCS < 10.
Atagi S. [32]	Patients older than 70 years. Randomized, controlled, phase 3 trial: chemoradiotherapy (concurrent low–dose carboplatin) or radiotherapy alone,	Some elderly should be considered for chemoradiotherapy due to benefit of decreased mortality (HR = 0.68, *p* = 0.0179). Chemoradiotherapy was associated with more rate of grade 3–4 hematological toxicity.

## Data Availability

Data may be available upon reasonable request and with permission of the National Research Institute of Oncology in Warsaw (Poland).

## References

[1] Non-small Cell Lung Cancer Collaborative Group (1995). Chemotherapy in non-small cell lung cancer: A meta-analysis using updated data on individual patients from 52 randomised clinical trials. BMJ.

[2] Auperin A., Le Pechoux C., Rolland E., Curran W.J., Furuse K., Fournel P., Belderbos J., Clamon G., Ulutin H.C., Paulus R. (2010). Meta-analysis of concomitant versus sequential radiochemiotherapy in locally advanced non-small-cell lung cancer. J. Clin. Oncol..

[3] Antonia S.J., Villegas A., Daniel D., Vicente D., Murakami S., Hui R., Kurata T., Chiappori A., Lee K.H., de Wit M. (2018). Overall Survival with Durvalumab after Chemoradiotherapy in Stage III NSCLC. N. Engl. J. Med..

[4] Gray J.E., Villegas A., Daniel D., Vicente D., Murakami S., Hui R., Kurata T., Chiappori A., Lee K.H., Cho B.C. (2020). Three-Year Overall Survival with Durvalumab after chemoradiotherapy in stage III NSCLC- Update from PACIFIC. J. Thorac. Oncol..

[5] Pentheroudakis G., ESMO Guidelines Committee (2020). Recent eUpdate to the ESMO Clinical Practice Guidelines on early and locally advanced non-small-cell lung cancer (NSCLC). Ann. Oncol..

[6] Park K., Vansteenkiste J., Lee K.H., Pentheroudakis G., Zhou C., Prabhash K., Seto T., Voon P.J., Tan D.S.W., Yang J.C.H. (2020). Pan-Asian adapted ESMO Clinical Practice Guidelines for the management of patients with locally-advanced unresectable non-small-cell lung cancer: A KSMO-ESMO initiative endorsed by CSCO, ISMPO, JSMO, MOS, SSO and TOS. Ann. Oncol..

[7] Cardenal F., Nadal E., Jové M., Faivre-Finn C. (2015). Concurrent systemic therapy with radiotherapy for the treatment of poor-risk patients with unresectable stage III non-small-cell lung cancer: A review of the literature. Ann. Oncol..

[8] De Ruysscher D., Botterweck A., Dirx M., Pijls-Johannesma M., Wanders R., Hochstenbag M., Dingemans A.M., Bootsma G., Geraedts W., Simons J. (2009). Eligibility for concurrent chemotherapy and radiotherapy of locally advanced lung cancer patients: A prospective, population-based study. Ann. Oncol..

[9] Atagi S., Kawahara M., Tamura T., Noda K., Watanabe K., Yokoyama A., Sugiura T., Senba H., Ishikura S., Ikeda H. (2005). Standard thoracic radiotherapy with or without concurrent daily low-dose carboplatin in elderly patients with locally advanced non-small cell lung cancer: A Phase III trial of the Japan Clinical Oncology Group (JCOG9812). Jpn. J. Clin. Oncol..

[10] Stinchcombe T.E., Zhang Y., Vokes E.E., Schiller J.H., Bradley J.D., Kelly K., Curran W.J., Schild S.E., Movsas B., Clamon G. (2017). Pooled analysis of individual patient data on concurrent chemoradiotherapy for stage III non-small-cell lung cancer in elderly patients compared with younger patients who participated in US National Cancer Institute cooperative group studies. J. Clin. Oncol..

[11] Miller E.D., Fisher J.L., Haglund K.E., Grecula J.C., Xu-Welliver M., Bertino E.M., He K., Shields P.G., Carbone D.P., Williams T.M. (2018). The Addition of Chemotherapy to Radiation Therapy Improves Survival in Elderly Patients with Stage III Non-Small Cell Lung Cancer. J. Thorac. Oncol..

[12] Laine A.M., Westover K.D., Choy H. (2014). Radiation therapy as a backbone of treatment of locally advanced non-small cell lung cancer. Semin. Oncol..

[13] Maguire J., Khan I., McMenemin R., O’Rourke N., McNee S., Kelly V., Peedell C., Snee M. (2014). SOCCAR: A randomised phase II trial comparing sequential versus concurrent chemotherapy and radical hypofractionated radiotherapy in patients with inoperable stage III Non-Small Cell Lung Cancer and good performance status. Eur. J. Cancer.

[14] Walraven I., Damhuis R.A., Ten Berge M.G., Rosskamp M., van Eycken L., de Ruysscher D., Belderbos J.S.A. (2017). Treatment Variation of Sequential versus Concurrent Chemoradiotherapy in Stage III Non-Small Cell Lung Cancer Patients in the Netherlands and Belgium. Clin. Oncol..

[15] Firat S., Byhardt R.W., Gore E. (2002). Comorbidity and Karnofksy performance score are independent prognostic factors in stage III non-small-cell lung cancer: An institutional analysis of patients treated on four RTOG studies. Radiation Therapy Oncology Group. Int. J. Radiat. Oncol. Biol. Phys..

[16] Mohile S.G., Dale W., Somerfield M.R., Schonberg M.A., Boyd C.M., Burhenn P.S., Canin B., Cohen H.J., Holmes H.M., Hopkins J.O. (2018). Practical Assessment and Management of Vulnerabilities in Older Patients Receiving Chemotherapy: ASCO Guideline for Geriatric Oncology. J. Clin. Oncol..

[17] Locher C., Pourel N., Le Caer H., Berard H., Auliac J.B., Monnet I., Descourt R., Vergnenègre A., Lafay I.M., Greillier L. (2018). Impact of a comprehensive geriatric assessment to manage elderly patients with locally advanced non-small-cell lung cancers: An open phase II study using concurrent cisplatin-oral vinorelbine and radiotherapy (GFPC 08-06). Lung Cancer.

[18] Antonio M., Saldaña J., Linares J., Ruffinelli J.C., Palmero R., Navarro A., Arnaiz M.D., Brao I., Aso S., Padrones S. (2018). Geriatric assessment may help decision-making in elderly patients with inoperable, locally advanced non-small-cell lung cancer. Br. J. Cancer.

[19] Postmus P.E., Kerr K.M., Oudkerk M., Senan S., Waller D.A., Vansteenkiste J., Escriu C., Peters S., ESMO Guidelines Committee (2017). Early and locally advanced non-small-cell lung cancer (NSCLC): ESMO Clinical Practice Guidelines for diagnosis, treatment and follow-up. Ann. Oncol..

[20] Charlson M., Szatrowski T.P., Peterson J., Gold J. (1994). Validation of a combined comorbidity index. J. Clin. Epidemiol..

[21] Colinet B., Jacot W., Bertrand D., Lacombe S., Bozonnat M.C., Daurès J.P., Pujol J.L., oncoLR health network (2005). A new simplified comorbidity score as a prognostic factor in non-small-cell lung cancer patients: Description and comparison with the Charlson’s index. Br. J. Cancer.

[22] Lee J.H., Wu H.G., Kim H.J., Kim D.W., Lee S.H., Kim T.M., Kim Y.W., Heo D.S. (2012). Influence of Comorbidities on the Efficacy of Radiotherapy with or without Chemotherapy in Elderly Stage III Non-small Cell Lung Cancer Patients. Cancer Res. Treat..

[23] Blanco R., Maestu I., de la Torre M.G., Cassinello A., Nuñez I. (2015). A review of the management of elderly patients with non-small-cell lung cancer. Ann. Oncol..

[24] Coate L.E., Massey C., Hope A., Sacher A., Barrett K., Pierre A., Leighl N., Brade A., de Perrot M., Waddell T. (2011). Treatment of the elderly when cure is the goal: The influence of age on treatment selection and efficacy for stage III non-small cell lung cancer. J. Thorac. Oncol..

[25] Eberhardt W.E., De Ruysscher D., Weder W., Le Péchoux C., De Leyn P., Hoffmann H., Westeel V., Stahel R., Felip E., Peters S. (2015). 2nd ESMO Consensus Conference in Lung Cancer: Locally advanced stage III non-small-cell lung cancer. Ann. Oncol..

[26] Hayakawa K., Mitsuhashi N., Katano S., Saito Y., Nakayama Y., Sakurai H., Akimoto T., Hasegawa M., Yamakawa M., Niibe H. (2001). High-dose radiation therapy for elderly patients with inoperable or unresectable non-small cell lung cancer. Lung Cancer.

[27] Dillman R.O., Seagren S.L., Propert K.J., Guerra J., Eaton W.L., Perry M.C., Carey R.W., Frei E.F., Green M.R. (1990). A randomized trial of induction chemotherapy plus high-dose radiation versus radiation alone in stage III non-small-cell lung cancer. N. Engl. J. Med..

[28] Pallis A.G., Gridelli C., van Meerbeeck J.P., Greillier L., Wedding U., Lacombe D., Welch J., Belani C.P., Aapro M. (2010). EORTC Elderly Task Force and Lung Cancer Group and International Society for Geriatric Oncology (SIOG) experts’ opinion for the treatment of non-small-cell lung cancer in an elderly population. Ann. Oncol..

[29] Schild S.E., Stella P.J., Geyer S.M., Bonner J.A., McGinnis W.L., Mailliard J.A., Brindle J., Jatoi A., Jett J.R., North Central Cancer Treatment Group (2003). The outcome of combined-modality therapy for stage III non-small-cell lung cancer in the elderly. J. Clin. Oncol..

[30] Jalal S.I., Riggs H.D., Melnyk A., Richards D., Agarwala A., Neubauer M., Ansari R., Govindan R., Bruetman D., Fisher W. (2012). Updated survival and outcomes for older adults with inoperable stage III non-small-cell lung cancer treated with cisplatin, etoposide, and concurrent chest radiation with or without consolidation docetaxel: Analysis of a phase III trial from the Hoosier Oncology Group (HOG) and US Oncology. Ann. Oncol..

[31] Semrau S., Bier A., Thierbach U., Virchow C., Ketterer P., Klautke G., Fietkau R. (2007). 6-year experience of concurrent radiochemotherapy with vinorelbine plus a platinum compound in multimorbid or aged patients with inoperable non-small cell lung cancer. Strahlenther. Onkol..

[32] Atagi S., Kawahara M., Yokoyama A., Okamoto H., Yamamoto N., Ohe Y., Sawa T., Ishikura S., Shibata T., Fukuda H. (2012). Thoracic radiotherapy with or without daily low-dose carboplatin in elderly patients with non-small-cell lung cancer: A randomised, controlled, phase 3 trial by the Japan Clinical Oncology Group (JCOG0301). Lancet Oncol..

[33] Movsas B., Scott C., Sause W., Byhardt R., Komaki R., Cox J., Johnson D., Lawton C., Dar A.R., Wasserman T. (1999). The benefit of treatment intensification is age and histology-dependent in patients with locally advanced non-small cell lung cancer (NSCLC): A quality-adjusted survival analysis of radiation therapy oncology group (RTOG) chemoradiation studies. Int. J. Radiat. Oncol. Biol. Phys..

[34] Gaspar L.E., Chansky K., Albain K.S., Vallieres E., Rusch V., Crowley J.J., Livingston R.B., Gandara D.R. (2005). Time from treatment to subsequent diagnosis of brain metastases in stage III non-small-cell lung cancer: A retrospective review by the Southwest Oncology Group. J. Clin. Oncol..

[35] De Leyn P., Vansteenkiste J., Lievens Y., Van Raemdonck D., Nafteux P., Decker G., Coosemans W., Decaluwé H., Moons J., Lerut T. (2009). Survival after trimodality treatment for superior sulcus and central T4 non-small cell lung cancer. J. Thorac. Oncol..

[36] Goldstraw P., Crowley J., Chansky K., Giroux D.J., Groome P.A., Rami-Porta R., Postmus P.E., Rusch V., Sobin L., International Association for the Study of Lung Cancer International Staging Committee (2007). The IASLC Lung Cancer Staging Project: Proposals for the revision of the TNM stage groupings in the forthcoming (seventh) edition of the TNM classification of malignant tumours. J. Thorac. Oncol..

[37] Brunelli A., Charloux A., Bolliger C.T., Rocco G., Sculier J.P., Varela G., Licker M., Ferguson M.K., Faivre-Finn C., Huber R.M. (2009). ERS/ESTS clinical guidelines on fitness for radical therapy in lung cancer patients (surgery and chemo-radiotherapy). Eur. Respir. J..

[38] Stinchcombe T.E., Bogart J.A. (2012). Novel approaches of chemoradiotherapy in unresectable stage IIIA and stage IIIB non-small cell lung cancer. Oncologist.

[39] Zaborowska-Szmit M., Krzakowski M., Kowalski D.M., Szmit S. (2020). Cardiovascular Complications of Systemic Therapy in Non-Small-Cell Lung Cancer. J. Clin. Med..

[40] Łazar-Poniatowska M., Bandura A., Dziadziuszko R., Jassem J. (2021). Concurrent chemoradiotherapy for stage III non-small-cell lung cancer: Recent progress and future perspectives (a narrative review). Transl. Lung Cancer Res..

[41] Bezjak A., Temin S., Franklin G., Giaccone G., Govindan R., Johnson M.L., Rimner A., Schneider B.J., Strawn J., Azzoli C.G. (2015). Definitive and Adjuvant Radiotherapy in Locally Advanced Non-Small-Cell Lung Cancer: American Society of Clinical Oncology Clinical Practice Guideline Endorsement of the American Society for Radiation Oncology Evidence-Based Clinical Practice Guideline. J. Clin. Oncol..

[42] Faivre-Finn C., Vicente D., Kurata T., Planchard D., Paz-Ares L., Vansteenkiste J.F., Spigel D.R., Garassino M.C., Reck M., Senan S. (2021). Four-Year Survival with Durvalumab After Chemoradiotherapy in Stage III NSCLC-an Update From the PACIFIC Trial. J. Thorac. Oncol..

